# Supplementary material for: Diagnostic Value of Neutrophil-to-Lymphocyte, Platelet-to-Lymphocyte, and Monocyte-to-Lymphocyte Ratios for the Assessment of Rheumatoid Arthritis in Patients with Undifferentiated Inflammatory Arthritis
Source: Diagnostics (Basel). 2022 Jul 13;12(7):1702. doi: 10.3390/diagnostics12071702 (PMC9317908; doi:10.3390/diagnostics12071702)
Supplement: Supplementary file 1 [file diagnostics-12-01702-s001.zip › diagnostics-1767448-supplementary.pdf]

Supplement Table S1. Baseline clinical characteristics between patients with "clinical rheumatoid arthritis" and "non-clinical rheumatoid arthritis"

	Clinical RA (n = 65)	Non-clinical RA (n = 136)	*p* value
Age, years, mean ± SD	59.84±14.7	58.3±12	0.478
NLR, median, (IQR)	2.34 (1.60-3.84)	1.74 (1.29-2.41)	<0.001
PLR, median, (IQR)	153.58 (110.65-232.15)	132.43 (110.05-176.84)	0.034
MLR, median, (IQR)	0.28 (0.20-0.42)	0.21 (0.16-0.28)	<0.001
CRP, mg/dL, median, (IQR)	0.48 (0.11-1.49)	0.05 (0.03-0.16)	<0.001
ESR, mm/hr, median, (IQR)	33 (16-60)	12 (5-22)	<0.001
APR score, median, (IQR)	1 (1-1)	0 (0-1)	<0.001
Sx duration, month, median, (IQR)	12 (7-24)	11 (4-11)	0.003
Sx score, median, (IQR)	1 (1-1)	1 (0-1)	0.058
SJC, n, median, (IQR)	2 (1-3)	1 (1-1)	<0.001
TJC, n, median, (IQR)	2 (2-4)	2 (1-4)	0.024
Small joint involvement, median, (IQR)	2 (2-4)	1 (1-2)	0.001
Large joint involvement, n, median, (IQR)	0 (0-1)	0(0-1)	0.886
Joint score, median, (IQR)	2 (2-3)	2 (2-2)	0.022
RF, IU/mL, median, (IQR)	42.6 (12.4-112.9)	8.5 (7.0-11.7)	<0.001
RF positivity, n (%)	46 (70.8)	23 (16.9)	<0.001
Anti-CCP, U/mL, median, (IQR)	41.9 (5.56-200.0)	0.4 (0.2-0.8)	<0.001
Anti-CCP positivity, n (%)	50 (76.9)	5 (3.7)	<0.001
Antibody score, median, (IQR)	3 (2-3)	0 (0-0)	<0.001
Classification score, median, (IQR)	7 (6-7)	3 (2-4)	<0.001

RA rheumatoid arthritis, SD standard deviation, IQR interquartile range, NLR neutrophil-to-lymphocyte ratio, PLR platelet-to-lymphocyte ratio, MLR monocyte-to-lymphocyte ratio, CRP C-reactive protein, ESR erythrocyte sedimentation rate, APR acute-phase response, Sx symptom, SJC swollen joint count, TJC tender joint count, RF rheumatoid factor, CCP cyclic citrullinated protein.

Supplement Table S2. Baseline clinical characteristics between patients with "disease modifying anti-rheumatic drugs rheumatoid arthritis" and "non- disease modifying anti-rheumatic drugs rheumatoid arthritis"

	DMARDs RA (n = 63)	Non-DMARDs RA (n = 138)	*p* value
Age, years, mean ± SD	59.6±14.5	58.5±12.1	0.550
NLR, median, (IQR)	2.35 (1.61-3.91)	1.75 (1.28-2.40)	<0.001
PLR, median, (IQR)	153.58 (111.46-232.34)	132.43 (109.93-178.04)	0.023
MLR, median, (IQR)	0.28 (0.20-0.43)	0.21 (0.16-0.28)	<0.001
CRP, mg/dL, median, (IQR)	0.54 (0.12-1.61)	0.05 (0.03-0.16)	<0.001
ESR, mm/hr, median, (IQR)	34 (16-62)	12.5 (6-22)	<0.001
APR score, median, (IQR)	1 (1-1)	0 (0-1)	<0.001
Sx duration, month, median, (IQR)	12 (6-24)	11 (4-12)	0.003
Sx score, median, (IQR)	1 (1-1)	1 (0-1)	0.082
SJC, n, median, (IQR)	2 (1-3)	1 (1-1)	<0.001
TJC, n, median, (IQR)	2 (2-4)	2 (1-4)	0.02
Small joint involvement, n, median, (IQR)	2 (2-4)	1 (1-2)	0.001
Large joint involvement, n, median, (IQR)	0 (0-1)	0 (0-1)	0.934
Joint score, median, (IQR)	2 (2-3)	2 (2-2)	0.036
RF, IU/mL, median, (IQR)	42.6 (12.5-118.7)	8.5 (7.0-11.8)	<0.001
RF positivity, n (%)	45 (71.4)	24 (17.4)	<0.001
Anti-CCP, U/mL, median, (IQR)	41.9 (5.6-200.0)	0.4 (0.2-0.8)	<0.001
Anti-CCP positivity, n (%)	49 (77.8)	6 (4.3)	<0.001
Antibody score, median, (IQR)	3 (2-3)	0 (0-0)	<0.001
Classification score, median, (IQR)	7(6-7)	3 (3-4)	<0.001

DMARDs disease modifying anti-rheumatic drugs, RA rheumatoid arthritis, SD standard deviation, IQR interquartile range, NLR neutrophil-to-lymphocyte ratio, PLR platelet-to-lymphocyte ratio, MLR monocyte-to-lymphocyte ratio, CRP C-reactive protein, ESR erythrocyte sedimentation rate, APR = acute-phase response, Sx symptom, SJC swollen joint count, TJC tender joint count, RF rheumatoid factor, CCP cyclic citrullinated protein.

Supplement Table S3. Baseline clinical characteristics between patients with "classified rheumatoid arthritis" and "non-classified rheumatoid arthritis"

	Classified RA (n = 61)	Non-classified RA (n = 140)	*p* value
Age, years, mean ± SD	60.2±14.8	58.3±11.9	0.374
NLR, median, (IQR)	2.23 (1.51-3.71)	1.83 (1.30-2.52)	0.006
PLR, median, (IQR)	153.91 (110.21-232.15)	132.82 (110.05-183.9)	0.069
MLR, median, (IQR)	0.27 (0.20-0.42)	0.22 (0.17-0.28)	<0.001
CRP, mg/dL, median, (IQR)	0.42 (0.09-1.28)	0.05 (0.03-0.23)	<0.001
ESR, mm/hr, median, (IQR)	33 (18-58)	12 (6-22)	<0.001
APR score, median, (IQR)	1 (1-1)	0 (0-1)	<0.001
Sx duration, month, median, (IQR)	12 (7-24)	11 (4-21)	0.006
Sx score, median, (IQR)	1 (1-1)	1 (0-1)	0.023
SJC, n, median, (IQR)	2 (2-3)	1 (1-1)	<0.001
TJC, n, median, (IQR)	3 (2-4)	2 (1-4)	<0.001
Small joint involvement, n, median, (IQR)	2 (2-4)	1 (0-2)	<0.001
Large joint involvement, n, median, (IQR)	0 (0-1)	0 (0-1)	0.596
Joint score, median, (IQR)	2 (2-3)	2 (2-2)	<0.001
RF, IU/mL, median, (IQR)	51.2 (19.8-125.9)	8.4 (7.0-10.8)	<0.001
RF positivity, n (%)	52 (85.2)	17 (12.1)	<0.001
Anti-CCP, U/mL, median, (IQR)	48.8 (12.1-200.0)	0.4 (0.2-0.8)	<0.001
Anti-CCP positivity, n (%)	49 (80.3)	6 (4.3)	<0.001
Antibody score, median, (IQR)	3 (3-3)	0 (0-0)	<0.001
Classification score, median, (IQR)	7 (6-7)	3 (2-4)	<0.001

RA rheumatoid arthritis, SD standard deviation, IQR interquartile range, NLR neutrophil-to-lymphocyte ratio, PLR platelet-to-lymphocyte ratio, MLR monocyte-to-lymphocyte ratio, CRP C-reactive protein, ESR erythrocyte sedimentation rate, APR acute-phase response, Sx symptom, SJC swollen joint count, TJC tender joint count, RF rheumatoid factor, CCP cyclic citrullinated protein.
